# Analysis of a Systemic Inflammatory Biomarker in Advanced Bile Tract Carcinoma Treated with Anti-PD-1 Therapy: Prognostic and Predictive Significance of Lung Immune Prognostic Index Score

**DOI:** 10.1155/2022/1427779

**Published:** 2022-03-17

**Authors:** Yuting Pan, Hanyan Si, Ru Jia, Guochao Deng, Huan Yan, Mengjia Fan, Miaomiao Gou, Shiyun Chen, Nan Zhang, Yue Shi, Niansong Qian, Guanghai Dai

**Affiliations:** ^1^Medical Oncology Department, The First Medical Center, Chinese People's Liberation Army General Hospital, Beijing 100853, China; ^2^Chinese People's Liberation Army Medical School, Beijing 100853, China; ^3^The Hainan Medical Center, Chinese PLA General Hospital, Sanya 572000, China; ^4^The Second School of Clinical Medicine, Southern Medical University, Guangzhou 510220, Guangdong Province, China

## Abstract

**Background:**

The application of immunotherapy is gradually increasing in advanced bile tract carcinoma (BTC), but only some patients could benefit from it. Validated biomarkers can screen out the beneficiaries. Therefore, the objective of this research is aimed at exploring the predictive value of lung immune prognostic index (LIPI) in advanced BTC patients receiving immunotherapy.

**Methods:**

This study was conducted on 110 BTC patients. The cut-off value of the derived neutrophil-to-lymphocyte (dNLR) ratio was obtained by the ROC curves to predict the tumor progression rate at the 6^th^ month. The high levels of dNLR (≥the cut-off value) and lactate dehydrogenase (≥the upper limit of normal) were considered to be two risk factors for LIPI. Based on these two risk factors, patients were categorized into 3 groups based on risk factors: 0 for the good group, 1 for the intermediate group, and 2 for the poor group. Due to the limited number of patients in the poor group, it was integrated into the intermediate group to be the intermediate/poor group. Finally, the subjects were divided into two groups: LIPI-good and LIPI-intermediate/poor.

**Results:**

The results shed light on the 110 BTC patients' LIPI in advanced BTC patients receiving immunotherapy, indicating that the cut-off value of dNLR was 1.74. According to the risk stratification, 38 (34.5%) patients had a good LIPI score, whereas the LIPI score was intermediate/poor in 72 (65.5%). In addition, patients with good LIPI were related to longer progression-free survival (PFS) and overall survival (OS), compared to those with intermediate/poor LIPI (12.17 months vs. 3.17 months; 20.2 months vs. 8.7 months). According to multivariate analysis, the intermediate/poor LIPI group was independently correlated with over 2.3 times greater risk of tumor progression (HR = 2.301; 95% CI, 1.395-3.796; *P* = 0.001) and over 1.8 times greater risk of death (HR = 1.877; 95% CI, 1.076-3.275; *P* = 0.027) than the good group. Moreover, the result also revealed that there were significant differences of DCR for patients of the good group and the intermediate/poor group (86.8% vs. 65.3%; *P* = 0.012).

**Conclusion:**

Finally, this study verifies, for the first time, that LIPI is an independent factor affecting the survival and clinical efficacy of advanced BTC patients receiving immunotherapy. It may be difficult for patients with intermediate/poor LIPI to benefit from immunotherapy.

## 1. Introduction

In developed countries, biliary tract cancer (BTC) is an uncommon but clinically aggressive cancer, consisting of intrahepatic and extrahepatic cholangiocarcinoma (CCA) and gallbladder cancers [1]. CCA is a malignant tumor, derived from bile duct epithelial cells [2]. The development of Multidisciplinary Treatment (MDT) (i.e., radiotherapist, medical oncologist, and hepatobiliary surgeon) enables CCA patients to obtain the best treatment plan [2]. Recently, the incidence of BTC has increased year by year [3]. Surgery is the main treatment for BTC, but only 20% of BTC patients who are in early stages are candidates [4]. Although BTC is not as infamous as the king of carcinoma, pancreatic cancer, it is still a refractory, high-risk malignant tumor with a high mortality rate. BTC is prone to relapse and metastasis despite early surgical intervention [5]. Chemotherapy is the standard treatment of BTC, but its effect is limited [6]. The established first-line systemic treatment for advanced BTC, albumin-bound paclitaxel combined with gemcitabine and cisplatin (GP), reported that its overall survival (OS) does not exceed one year [7]. Advanced BTC patients did not benefit from targeted therapy reported by some studies [8]. Therefore, immune checkpoint inhibitors (ICIs) have not only become the established first-line systemic treatment for some solid tumors but also bring long-term clinical benefits to patients [9]. ICIs can provide advanced BTC patients durable clinical efficacy with a controllable safety profile, as reported in some studies [10, 11]. Although immunotherapy brings promise to BTC patients, only some patients can benefit from it. Therefore, finding effective and practical biomarkers that can predict the prognosis of immunotherapy is crucial in prolonging life and improving the quality of life for BTC patients. In addition, the combination of multiple indicators is more accurate than a single biomarker in screening people who may benefit from immunotherapy, and it can also provide more specific information in detecting potential subgroups that may benefit from such therapy.

Lung immune prognostic index (LIPI) consists of lactate dehydrogenase (LDH) levels and derived neutrophil-to-lymphocyte (dNLR) ratio. As an effective and economical biomarker, LIPI is indicative of immunotherapeutic efficiency and survival outcomes. Since Mezquita et al. initially reported that LIPI serves as an independent indicator correlated with the outcomes of non-small-cell lung cancer (NSCLC) receiving immunotherapy, numerous researchers validated its value in forecasting the prognosis of other solid tumor patients after immunotherapy [12]. However, whether LIPI is an independent factor affecting the survival and clinical efficacy of advanced BTC patients receiving immunotherapy remains controversial. Thus, this study aimed at exploring the clinical value of LIPI in predicting the prognostic outcomes of advanced BTC patients following ICI treatment.

## 2. Materials and Methods

### 2.1. Study Population

Institutional review board approval was acquired to review medical records at the Chinese People's Liberation Army General Hospital (approval number: S2019-136-01). All the patients involved were diagnosed with BTC at stage IV and received ICI treatment in the Senior Department of Oncology, Chinese PLA General Hospital, from September 2015 to April 2021. The inclusion criteria were set as the following: (1) patients detected with measurable lesions, (2) patients conducted blood routine and blood biochemistry tests within one week before ICI administration, and (3) patients continuously received at least two rounds of ICI treatment. Patients failing to provide imaging data for comparing the efficacy of ICIs before and after treatment were excluded. As a result, a total of 100 patients were considered eligible for this cohort study. Clinical parameters of those BTC patients from their medical records were collected, including sex, age, Eastern Cooperative Oncology Group performance status scores (ECOG PS), smoking history, smoking exposure, history of diabetes, tumor type, liver metastasis, number of metastatic sites, line of treatment with ICIs, ICI agent, immunotherapy scheme, and treatment-related adverse events (TRAEs). Meanwhile, we analyzed their blood routine parameters such as white blood cell count (WBC) and absolute neutrophil count (ANC) 7 days before implementing immunotherapy to obtain dNLR (neutrophil/(white blood cell-neutrophil)) value as well as blood biochemical data to obtain LDH value.

### 2.2. Treatment Regimens

The types and doses of ICIs were as follows: (1) sintilimab was injected intravenously 200 mg once every 3 weeks; (2) toripalimab was injected intravenously 240 mg once every 3 weeks; (3) pembrolizumab was injected intravenously at a recommended dose rate of 3 mg/kg, administered once every 3 weeks; (4) nivolumab was injected intravenously at a recommended dosage rate of 2 mg/kg, administered once every 2 weeks. The first imaging evaluation of nivolumab was carried out 2-4 weeks after the 3^rd^ intravenous injection. However, the evaluation of toripalimab, sintilimab, and pembrolizumab was carried out 3-5 weeks after the 2^nd^ intravenous injection. Four treatment methods were used in this study, including ICIs combined with antiangiogenesis therapy, ICIs combined with chemotherapy, and ICIs combined with chemotherapy and antiangiogenic therapy. Antiangiogenic drugs involve lenvatinib (8-12 mg, orally administrated once a day), apatinib (850 mg, orally administrated 30 min after a meal, once a day), and bevacizumab (5 mg/kg body weight, once every two weeks; or 7.5 mg/kg body weight, once every 3 weeks).

The chemotherapy regimens include (1) GS regimen: tiggio (40-60 mg, 2 times a day, orally administrated after breakfast and dinner, 14 days in a row followed by 7 days of interval) and gemcitabine (1250 mg/m^2^ on the first day of each cycle, intravenous instillation); (2) GP regimen: cisplatin (75 mg/m^2^) and gemcitabine (1250 mg/m^2^, intravenous administration on the first day); (3) AS regimen: tiggio (40-60 mg, 2 times a day, orally administrated after breakfast and dinner, 14 days in a row followed by 7 days of interval, use it on the first day of each cycle); (4) GMOX regimen: oxaliplatin (130 mg/m^2^) combined with gemcitabine (1250 mg/m^2^, intravenously administrated on the first day); (5) AX regimen: capecitabine (1000 mg/m^2^, orally administrated twice a day, after breakfast and dinner, 14 consecutive in a row followed by 7 days of interval) conjugated albumin paclitaxel (260 mg/m^2^ intravenous injection the first day of each cycle); and (6) others. All plans were chosen based on the patient's pathological stage and general health conditions, and all patients signed informed consent for treatment.

### 2.3. Assessment

For efficacy evaluation, the disease control rate (DCR) and the overall response rate (ORR) is termed as the percentage of patients with complete response (CR), partial response (PR), and stable disease (SD) and the percentage of patients with CR and PR, respectively. For prognosis analysis, OS and progression-free survival (PFS) are the time from the beginning of immunotherapy to death and the time between the onset of ICIs and the progression or death of the tumor, respectively.

### 2.4. Statistical Analysis

SPSS 26.0 software was used to perform all statistical analyses. Data were summarized as the minimum-maximum range and median for nonnormally distributed continuous variables. Based on the values of *α* (*α* = 0.05) and *β* (*β* = 0.8), the expected median OS (mOS) of the good LIPI group (mOS = 20 months) and the intermediate/poor LIPI group (mOS = 10 months), we evaluated the number of the sample size of our retrospective cohort study. The specific sample size is shown in attached file 1. Data were reported as percentages and counts for categorical variables. The receiver operating characteristic (ROC) curves were applied to clarify the best cut-off value of dNLR. *χ*^2^ or Fisher's exact test was carried out to evaluate the relationship between clinical response and LIPI of BTC patients. The survival curve was depicted by Kaplan–Meier analysis. Logistic regression models and Cox proportional-hazard models were applied to assess the prognostic values of LIPI for DCR and survival, respectively. *P* values less than 0.05 (*P* < 0.05) were considered statistically significant.

## 3. Results

### 3.1. Baseline Characteristics

A total of 110 advanced BTC patients receiving ICIs were included. The clinical features of the patients were provided in the following (Table 1). The median age of advanced BTC patients receiving ICIs was 59 years old. Patients were predominantly male (60.9%), no history of smoking (67.3%), short history of smoking exposure (smoking exposure history is less than or equal to 30 years in 79.1% of cases), ECOG PS of 0-1 (94.5%), the tumor type of CC (80%), no hepatic metastasis (58.2%), and had fewer organ metastasis (the number of metastatic sites of 0-1 in 64.5% of cases).

### 3.2. Treatment Characteristics

Of the 110 patients, 34 (30.9%) patients received nivolumab, 11 (10.0%) patients were treated with pembrolizumab, and 65 (59.1%) patients received other immunotherapy drugs; 79 (71.8%) patients were treated with the 1^st^ line ICIs, and 31 (28.2%) patients used ICIs after the 1^st^ line; 73 (66.4%) patients were treated with the combination of immunotherapy and chemotherapy, 9 (8.2%) patients were treated with the combination of immunotherapy and target therapy, 20 (18.2%) patients were treated with monotherapy of ICIs, and 8 (7.3%) patients were treated with the combination of immunotherapy, chemotherapy, and target therapy; 76 (69.1%) patients suffered from TRAEs (Table 1).

### 3.3. ROC Analysis and Grouping

**Figure 1:**
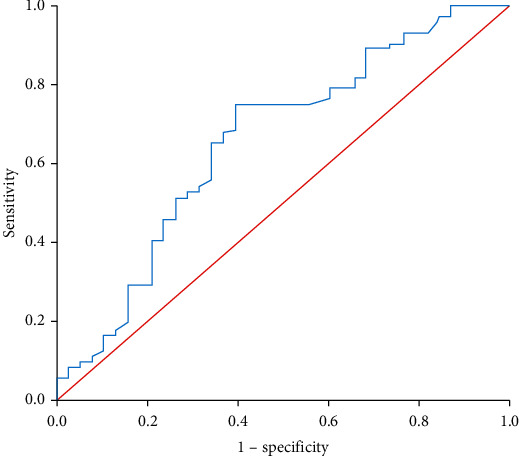
ROC curve of pretreatment dNLR in assessment of the tumor progression rate at 6^th^ month. ROC: receiver operator characteristic; dNLR: derived neutrophil-to-lymphocyte.

With dNLR before treatment as the test variable, and the tumor progression rate at the 6^th^ month as the state variable, the ROC curve of immunotherapy effect and dNLR level before treatment was generated. The area under the ROC curve was 0.657, which indicated a statistically significant difference (*P* = 0.007). The best cut-off value of dNLR was 1.74, and its corresponding sensitivity and specificity were 75% and 60.5%, respectively (Figure 1). The upper limit of LDH normal value was 245 U/L. The advanced BTC patients were divided into the good group and the intermediate/poor group according to the combination of LDH and the cut-off value of dNLR. The high levels of dNLR (≥1.74) and LDH (≥245 U/L) were considered to be two risk factors for the LIPI score system. Based on these two risk factors, patients were categorized into 3 groups based on risk factors: 0 for the good group, 1 for the intermediate group, and 2 for the poor group. Due to the limited number of patients in the poor group, it was integrated into the intermediate group to be the intermediate/poor group. Finally, according to the risk stratification, 38 (34.5%) patients had a good LIPI score, whereas the LIPI score was intermediate/poor in 72 (65.5%).

### 3.4. The Relationship between LIPI Groups and Response to ICI Treatment

The optimal efficacy of all BTC patients was evaluated in the study, and the results were as follows: 30 patients had progressive disease (PD), 2 patients had CR, 16 patients had PR, and 62 patients had SD. The ORR was 16.4% and DCR was 72.7% (Table 2). The results of logistic regression of disease control rate are summarized in Table 3. The multivariate logistic regression analysis showed that there was a discernable difference of DCR between BTC patients with intermediate/poor and good LIPI (65.3% vs. 86.8%; *P* = 0.012). The intermediate/poor group was associated with over 5 times greater risk of progressive disease than the LIPI-good group (OR = 5.217; 95% CI, 1.439-18.917). Additionally, we found that patients treated with the combination of immunotherapy and other therapies were associated with over 9 times greater risk of progressive disease than those treated with monotherapy of ICIs (OR = 9.548; 95% CI, 2.654-34.353; *P* = 0.001). No clear difference of ORR was observed between the intermediate/poor group and the good group (12.5% vs. 23.7%; *P* = 0.132).

### 3.5. The Prognostic Value of LIPI on Univariate Analysis

**Figure 2:**
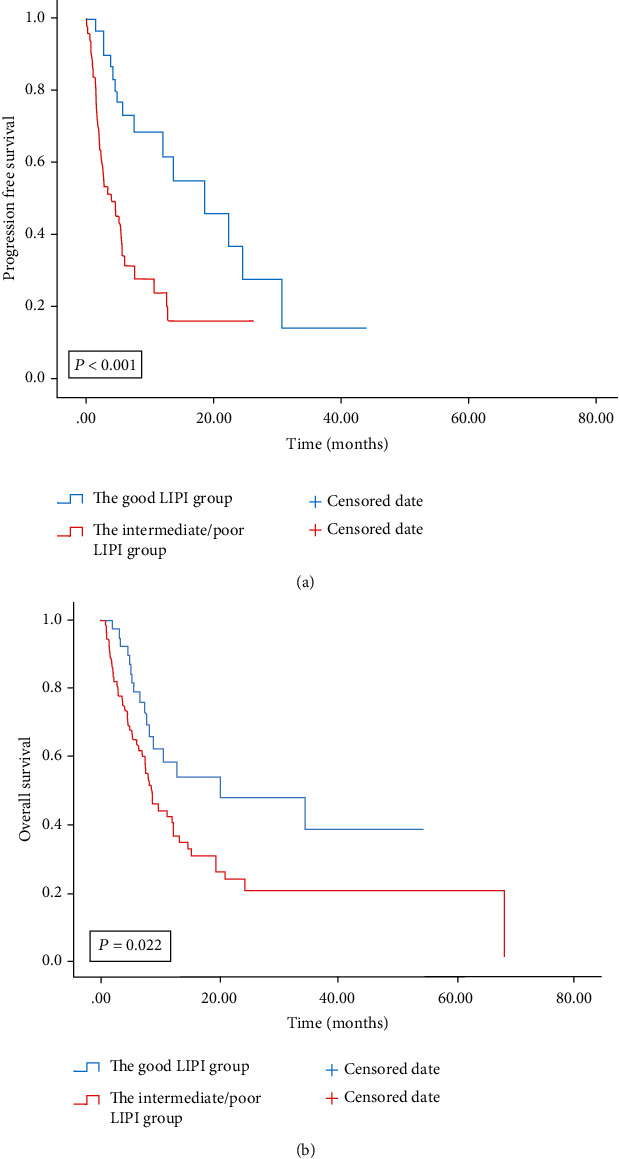
PFS (a) and OS (b) according to LIPI groups of patients with advanced BTC treated with the ICIs. PFS: progression-free survival; OS: overall survival; LIPI: lung immune prognostic index; BTC: biliary tract cancer; ICIs: immune checkpoint inhibitors.

Among the 110 advanced BTC patients, 66 (60%) patients died before the last follow-up date of June 14, 2021. Patients with a good LIPI were associated with longer PFS and OS, compared to those with intermediate/poor LIPI (12.17 months vs. 3.17 months; 20.2 months vs. 8.7 months) (Figures 2(a) and 2(b) and Table 4). In univariate analysis, patients with a good PS (ECOG PS of 0-1), with good LIPI, and treated with 1^st^ line ICIs were associated with improved PFS and OS (Table 5).

### 3.6. The Prognostic Value of LIPI on Multivariate Analysis

After we checked for hazard proportionality, the Cox regression multivariable approach was performed (Supplementary Figures 1a and 1b). Multivariate analysis revealed that patients treated with 1^st^ line ICIs were correlated with over 1.6 times greater risk of tumor progression (HR = 1.677; 95% CI, 1.055-2.665; *P* = 0.029) and patients that had poor PS (ECOG PS of ≥2) were correlated with over 5 times greater risk of death than patients that had a good PS (ECOG PS of 0-1) (HR = 5.383; 95% CI, 2.058-14.081; *P* = 0.001). Moreover, patients with the intermediate/poor LIPI were associated with more than twice the risk of tumor progression (HR = 2.301; 95% CI, 1.395-3.796; *P* < 0.001) and more than 1.8-fold increased risk of death than the good LIPI (HR = 1.877; 95% CI, 1.076-3.275; *P* = 0.027), respectively.

### 3.7. Association of the LIPI with Outcomes in Lines of Immunotherapy of 1 or in Subsequent Lines of Immunotherapy (≥2): Subgroup Analysis

**Figure 3:**
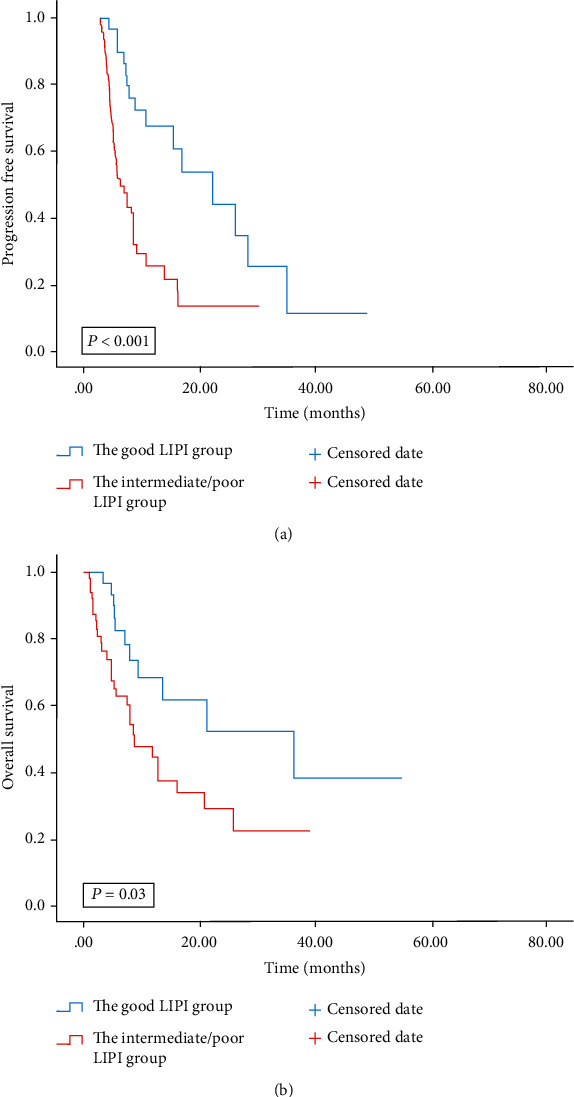
PFS (a) and OS (b) according to LIPI groups of patients with advanced BTC treated with the 1^st^ line ICIs. PFS: progression-free survival; OS: overall survival; LIPI: lung immune prognostic index; BTC: biliary tract cancer; ICIs: immune checkpoint inhibitors.

**Figure 4:**
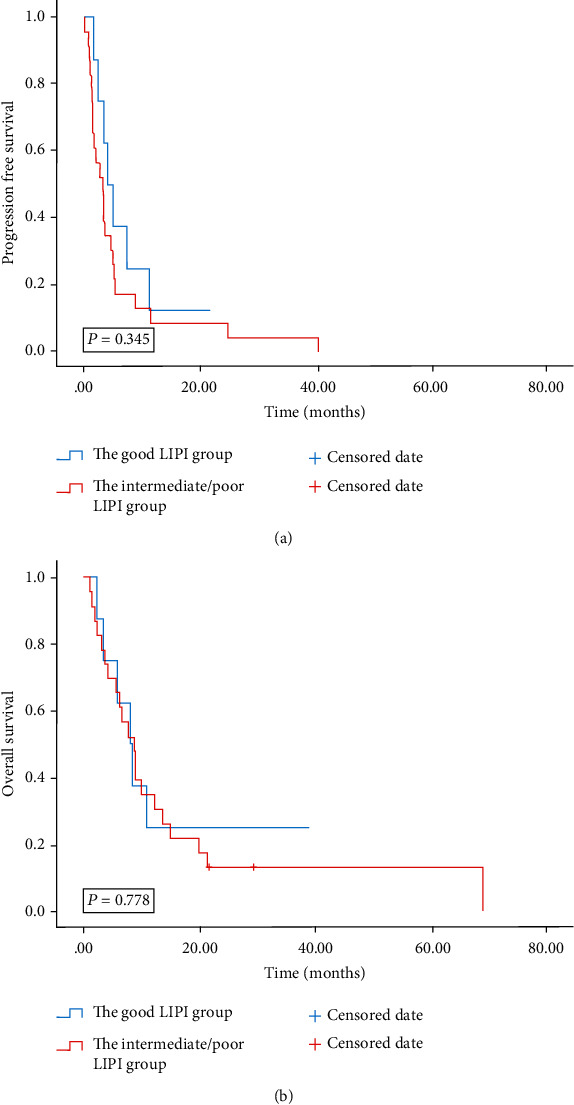
PFS (a) and OS (b) according to LIPI groups of patients with advanced BTC treated with the ICIs in subsequent lines. PFS: progression-free survival; OS: overall survival; LIPI: lung immune prognostic index; BTC: biliary tract cancer; ICIs: immune checkpoint inhibitors.

Multivariate analysis revealed that patients treated with the 1^st^ line ICIs were independently correlated with improved OS and PFS. Our study then conducted a subgroup analysis based on different lines of immunotherapy. Univariate analyses of the association of LIPI with outcomes in lines of immunotherapy of 1 are shown in Table 6. For the 79 patients treated with 1^st^ line ICIs, 49 patients were in the intermediate/poor LIPI group and 30 patients were in the good group. Patients with good LIPI had improved PFS and OS than those with intermediate/poor LIPI (12.93 months vs. 5.87 months; 34.43 months vs. 15.8 months) (Figures 3(a) and 3(b)). The intermediate/poor LIPI was correlated with over 2.1 times greater risk of death (HR = 2.147; 95% CI, 1.061-4.344; *P* = 0.03) and over 3 times greater risk of tumor progression (HR = 3.006; 95% CI, (1.601-5.642; *P* < 0.001) than the good LIPI. Univariate analyses of the association of LIPI with outcomes in subsequent lines of immunotherapy (≥2) are shown in Table 7. For the 31 patients treated with ICIs in subsequent lines, 23 patients were in the intermediate/poor LIPI group and 8 patients were in the good group. Patients with good and intermediate/poor LIPI did not show a clear difference in PFS and OS (3.9 months vs. 3.13 months; 8.7 months vs. 7.97 months; *P* = 0.345; *P* = 0.778) (Figures 4(a) and 4(b)).

## 4. Discussion

The usage of immunotherapy in the field of tumor treatment has been increasing annually worldwide, which has been proven to be widely used in a variety of solid tumors. However, immunotherapy drugs are expensive and prone to drug resistance and the occurrence of hyperprogressive disease (HPD). Therefore, looking for predictive indicators in the population that could benefit from immunotherapy could help lead to more precise and efficient immunotherapy treatment. The most widely studied markers for predicting the efficacy of immunotherapy are programmed death ligand-1 (*PD-L1*) expression, tumor mutational burden (TMB), and microsatellite steady-state (MSI). At present, the best biomarker for screening beneficial populations is *PD-L1*. Nevertheless, its screening capabilities are not consistent. The predictive value of PD-L1 on the efficacy of immunotherapy varies in different tumor types. It has predictive value for the prognosis of NSCLC, melanoma, and renal cell carcinoma, but it is controversial for colorectal cancer [13]. The optimum cut-off value of *PD-L1* positive also varies in different studies [14]. *PD-L1* expression in cells is not a fixed value, as it can be influenced by the collection time, the collection location, and the treatment plan [15]. TMB has emerged as an independent biomarker for immunotherapy. However, Horn et al. found that blood TMB (bTMB) is not associated with immune efficacy [16]. In addition, its application in clinical practice also has certain limitations. The TMB test relies on gene sequencing; thus, the cost is higher than the PD-L1 test. The storage time of specimens also has a certain influence on the judgment of TMB test results [16]. As a commonly used auxiliary diagnostic index, LDH level is an important and independent factor in predicting the prognosis of patients with malignant tumors receiving ICIs as shown by some studies [17]. A study on the use of ICI treatment for melanoma showed that high levels of LDH were associated with poor prognosis [17]. It is well established that immune performance and inflammatory response profoundly impact the long-term outcomes of cancer patients. Neutrophils, one type of the most vital and abundant leukocytes in the blood, are the first-line defense to protect the host from tissue damage and infection [18]. The number, subsets, and molecular characteristics of leukocytes have been analyzed in cancer patients as prognostic and predictive biomarkers for several decades [19]. Notoriously, the neutrophil-to-lymphocyte ratio has been proposed as an inflammatory biomarker elevated in patients with more advanced or aggressive diseases [19]. A blood-based liquid biopsy can capture circulating tumor cells and leukocytes, as well as circulating tumor-derived nucleic acids [19]. For the reason that a practical example in the frame of this thinking might be considering, within the field of inflammatory biomarkers, also integration with liquid biopsy and noninvasive tool [19]. Tumor-infiltrating lymphocytes are an indicator of the immune status in the tumor microenvironment, and the neutrophils are immunosuppressive. The reactive oxygen species released by neutrophil can damage DNA, which is related to the occurrence and development of tumors [20]. The neutrophil-to-lymphocyte ratio (NLR) can comprehensively reflect the immune status and inflammation of the tumor patients [21]. Existing studies show that high levels of NLR were associated with poor prognosis of lung cancer patients after immunotherapy [22]. dNLR is defined as the ratio between the pretreatment neutrophil and white blood cell minus neutrophil. The dNLR can reflect changes in the body's immune system, so it is more meaningful than NLR [12]. Regarding the criteria for judging the level of dNLR, large foreign clinical studies have selected 3 as the cut-off value [12]. Because of ethnic physiological differences, there were fewer patients with dNLR ≥ 3 in this study. If the cut-off value was set to be 3, there will be a big difference between the two groups of patients. Therefore, the cut-off value was determined by the ROC curve. The ROC curve was generated using the tumor progression status as the status variable and the dNLR level before treatment at the 6^th^ month of immunotherapy as the test variable. Finally, 1.74 was selected as the cut-off value of dNLR. Mezquita et al. and Kazandjian et al. found that in different treatment methods, LIPI was related to patients with PFS and OS [12, 23]. The high levels of dNLR (≥3) and LDH (≥245 U/L) were considered to be two risk factors for the LIPI score system in our study. Based on these two risk factors, BTC patients were categorized into 3 groups: the risk factor number for the “good” group was 0, for the “intermediate” group was 1, and for the “poor” group was 2 [12, 20, 24]. However, high level of dNLR (≥1.74) was considered a risk factor in our study. Some studies revealed that LIPI could be used as an effective and economical biomarker to predict immunotherapeutic efficiency and survival outcomes in gastric cancer, melanoma, renal cell carcinoma, and advanced hepatocellular carcinoma patients with ICIs [24–27]. The study first demonstrated the association of LIPI and clinical benefit, OS, and PFS in advanced BTC patients treated with ICIs. Multivariate analysis revealed that the good group was correlated independently with longer OS and PFS. The prognostic value of LIPI in advanced BTC was in close agreement with that of previous studies in some solid tumors [24–27]. Additionally, Chen et al., Benitez et al., and Hou et al. noticed that patients with a good PS (ECOG PS of 0-1) were also independently associated with PFS, OS, and DCR [24–27]. However, patients who had a poor PS (ECOG PS of ≥2) were independently associated with OS (HR = 5.383; 95% CI, 2.058-14.081; *P* = 0.001), not with PFS and DCR in our study. Chen et al. found that patients treated with the combination of immunotherapy and targeted therapy were independently associated with PFS (HR = 0.55; 95% CI, 0.32-0.94; *P* = 0.028) [25]. Furthermore, Hou et al. noticed that patients treated with the combination of immunotherapy and other therapies were associated with longer OS, with HRs of 0.58 (95% CI, 0.37-0.93; *P* = 0.024), and PFS, with HRs of 0.49 (95% CI, 0.30-0.81; *P* = 0.005) [27]. Nevertheless, we found that patients treated with the combination of immunotherapy and other therapies were associated with an over 9 times greater risk of tumor progression than those that used monotherapy (OR = 9.548; 95% CI, 2.654-34.353; *P* = 0.001). This result disagrees with the conclusion of Chen et al. and Hou et al. Mezquita et al. noticed that lines of immunotherapy were irrelevant to OS and PFS [12]. However, we found that patients treated with 1^st^ line ICIs were correlated with over 1.6 times greater risk of tumor progression (HR = 1.677; 95% CI, 1.055-2.665; *P* = 0.029). In addition, we found that two of the enrolled patients reached CR in the efficacy evaluation. Their common features include the following: (1) they are all women older than 58 years old, (2) they have liver metastases, and (3) they all use nivolumab combined with GP in the first line. Therefore, we speculate that older female patients may get good clinical benefits from nivolumab combined with GP in the first line. In recent years, peripheral blood inflammatory complex indexes such as NLR, platelet-lymphocyte ratio (PLR), dNLR, and hemoglobin (Hb) levels have demonstrated a potential prognostic biomarker for some solid tumors receiving ICIs [11, 28–30]. However, the mechanism of the correlation between these peripheral blood inflammatory complex indexes and the tumor prognosis is still unclear. Some studies have found that this was probably due to the tumor-immune microenvironment of patients [31, 32]. Patients with higher NLR typically have shorter OS and PFS, which may be related to the higher level of neutrophil-dependent inflammation in the immune microenvironment. dNLR can reflect the changes in the body's immune system as the increased dNLR is often caused by an increment of neutrophils or a decrease of lymphocytes. Increased number of neutrophils in the peripheral blood can promote tumor metastasis and growth by releasing inflammatory mediators [33]. On the other hand, decreased lymphocyte count will weaken the body's immune surveillance of tumor cells, which is conducive to tumor proliferation and metastasis. As a result, increased dNLR often indicates a poor prognosis in a variety of malignant tumors (pancreatic cancer, lymphoma, etc.). LDH is also a classic inflammatory marker and a manifestation of tumor burden in cancer patients. High levels of LDH are significantly correlated with worse PFS and OS. Alternatively, if it is not associated with other powerful clinical indicators (such as platelet count or NLR), the LDH level alone does not possess an independent prognostic significance [34, 35]. Therefore, the lower LIPI score the tumor patients have (i.e., the higher dNLR and LDH they get), the worse their prognosis will be. The reason of this phenomenon needs further study. LIPI, a practical and convenient prognostic marker, can be tested in almost all hospitals. It is expected to become a supplementary biomarker for advanced BTC with ICIs.

In the current study, some potential limitations should be noted. First, the primary limitation of this study is the small number of participants (*N* = 110). Due to the time and geographic limitations, this research investigated participants with a mixed population of intrahepatic advanced BTC, extrahepatic advanced BTC, and gallbladder cancer only from the same hospital. Additionally, a lack of comparison of LIPI score among the three cancers is another limitation which would reduce the reliability of the data analysis. Further study, therefore, is necessary to be conducted in advanced BTC treated with anti-PD-1 therapy for the study precision and reliability.

## 5. Conclusion

In conclusion, this study demonstrated that LIPI is independently correlated with the survival and clinical efficacy of advanced BTC patients receiving ICIs. However, the possibility of using LIPI as an effective and economic prognostic biomarker to selected patients, those who are best suited to receiving ICIs, needs further investigation in a larger prospective study.

## Figures and Tables

**Table 1:** Baseline clinical characteristics of BTC patients (*n*, %).

Characteristics	No. of patients (*n*, %)			
	Overall (*n* = 110)	The good LIPI (*n* = 38)	The intermediate LIPI (*n* = 56)	The poor LIPI (*n* = 16)
Median age (range), years	59 (24-91)	55.5 (24-70)	61 (42-80)	60 (44-91)
Sex				
Female	43 (39.1)	17 (44.7)	21 (37.5)	5 (36.1)
Male	67 (60.9)	21 (55.3)	35 (62.5)	11 (68.8)
Smoking history				
Yes	36 (32.7)	12 (31.6)	16 (28.6)	8 (50.0)
No	74 (67.3)	26 (68.4)	40 (71.4)	8 (50.0)
Smoking exposure				
>30 packs per year	23 (20.9)	10 (26.3)	8 (14.3)	5 (31.3)
≤30 packs per year	87 (79.1)	28 (73.7	48 (85.7)	11 (68.8)
History of diabetes				
Yes	19 (17.3)	6 (15.8)	10 (17.9)	3 (18.8)
No	91 (82.7)	32 (84.2)	46 (82.1)	13 (81.3)
Tumor type				
CC	88 (80.0)	32 (84.2)	45 (80.4)	11 (68.8)
GBC	22 (20.0)	6 (15.8)	11 (19.6)	5 (31.3)
Liver metastasis				
Present	46 (41.8)	18 (47.4)	18 (32.1)	10 (62.5)
Absent	64 (58.2)	20 (52.6)	38 (67.9)	6 (37.5
Number of metastatic sites				
≥2	39 (35.5)	14 (36.8)	17 (30.4)	8 (50.0)
<2	71 (64.5)	24 (63.2)	39 (69.6)	8 (50.0)
Lines of immunotherapy				
≥2	31 (28.2)	8 (21.1)	17 (69.6)	6 (37.5)
<2	79 (71.8)	30 (78.9)	39 (30.4)	10 (62.5)
ECOG PS				
≥2	6 (5.5)	1 (2.6)	2 (3.6)	3 (18.8)
0-1	104 (94.5)	37 (97.4)	54 (96.4)	13 (81.3)
PD-1 inhibition agent				
Nivolumab	34 (30.9)	14 (36.8)	16 (28.6)	4 (25.0)
Pembrolizumab	11 (10.0)	2 (5.3)	4 (7.1)	5 (31.3)
Others	65 (59.1)	22 (57.91)	36 (64.3)	7 (43.8)
ICIs combined with other therapies				
Monotherapy	20 (18.2)	8 (21.1)	9 (16.1)	3 (18.8)
Chemotherapy	73 (66.4)	25 (65.8)	37 (66.1)	11 (68.8)
Target therapy	9 (8.2)	4 (10.5)	5 (8.9)	0 (0)
Chemotherapy and target therapy	8 (7.3)	1 (2.6)	5 (8.9)	2 (12.5
Treatment-related adverse events				
Present	76 (69.1)	25 (65.8)	39 (69.6)	12 (75.0)
Absent	34 (30.9)	13 (34.2)	17 (30.4)	4 (25.0)

CC: GBC: ECOG PS: Eastern Cooperative Oncology Group performance status scores; PD-1: programmed cell death-1; ICIs: immune checkpoint inhibitors.

**Table 2:** Relationship between LIPI groups and response to anti-PD-1 treatment.

Best overall response	No. of patients (%)			*P* value
	Overall *n* = 110	The good LIPI *n* = 38	The intermediate/poor LIPI *n* = 72	
CR	2 (1.8)	1 (2.6)	1 (1.4)	1
PR	16 (14.5)	8 (21.1)	8 (11.1)	0.160
SD	62 (56.4)	24 (63.2)	38 (52.8)	0.297
PD	30 (27.3)	5 (13.2)	25 (34.7)	0.016^∗^
Objective response	18 (16.4)	9 (23.7)	9 (12.5)	0.132
Disease control rate	80 (72.7)	33 (86.8)	47 (65.3)	0.016∗

LIPI: lung immune prognostic index; PD-1: programmed cell death-1; CR: complete response; PR: partial response; SD: stable disease; PD: progressive disease.

**Table 3:** Logistic regression of disease control rate.

Characteristics	Univariate analysis		Multivariate analysis	
	OR (95% CI)	*P*	OR (95% CI)	*P*
Lines of immunotherapy				
<2	1 [ref.]		1 [ref.]	
≥2	0.250 (0.102-0.616)	0.003^∗^	0.401 (0.1441.120)	0.081
Combined with other therapies				
No	1 [ref.]	<0.001^∗^	1 [ref.]	0.001^∗^
Yes	7.975 (2.764-23.010)		9.548 (2.654-34.353)	
LIPI				
The good	1 [ref.]		1 [ref.]	
The intermediate/poor	3.511 (1.218-7.134)	0.002^∗^	5.217 (1.439-18.917)	0.012^∗^

LIPI: lung immune prognostic index.

**Table 4:** Efficacy and prognosis based on the LIPI groups.

LIPI classification		Response rate		OS (months)		PFS (months)	
		DCR (*n*, %)	OR (95% CI)	Median	HR (95% CI)	Median	HR (95% CI)
The good	(*n* = 38)	33 (86.8)	1 [reference]	20.2 (0.00-43.40)	1 [reference]	12.17 (4.29-20.05)	1 [reference]
The intermediate/poor	(*n* = 72)	47 (65.3)	5.217 (1.439-18.917)	8.7 (6.59-10.81)	1.877 (1.076-3.275)	3.17 (1.77-4.56)	2.301 (1.395-3.796)
*P* value		0.012^∗^		0.027^∗^		0.001^∗^	

PFS: progression-free survival; OS: overall survival; LIPI: lung immune prognostic index.

**(a):** 

Patient characteristics	Univariate analysis		Multivariate analysis	
OS	HR (95% CI)	*P*	HR (95% CI)	*P*
ECOG performance status				
0-1	1 [ref.]		1 [ref.]	
≥2	5.473 (2.099-14.266)	0.001^∗^	5.383 (2.058-14.081)	0.001^∗^
Lines of immunotherapy				
<2	1 [ref.]		1 [ref.]	
≥2	1.678 (1.021-2.758)	0.041^∗^	1.523 (0.919-2.526)	0.103
LIPI				
The good	1 [ref.]		1 [ref.]	
The intermediate/poor	1.892 (1.085-3.299)	0.024^∗^	1.877 (1.076-3.275)	0.027^∗^

**(b):** 

Patient characteristics	Univariate analysis		Multivariate analysis	
PFS	HR (95%CI)^a^	*P*	HR (95%CI)^a^	*P*
Lines of immunotherapy				
≥2	1 [ref.]		1 [ref.]	
<2	1.963 (1.247-3.091)	0.004^∗^	1.677 (1.055-2.665)	0.029^∗^
ECOG performance status				
≥2	1 [ref.]		1 [ref.]	
0-1	2.558 (1.022-6.404)	0.045^∗^	2.348 (0.927-5.946)	0.072
LIPI				
The good	1 [ref.]		1 [ref.]	
The intermediate/poor	2.524 (1.540-4.137)	<0.001^∗^	2.301 (1.395-3.796)	0.001^∗^

PFS: progression-free survival; OS: overall survival; LIPI: lung immune prognostic index; ECOG PS: Eastern Cooperative Oncology Group performance status scores.

**Table 6:** Univariate analyses of LIPI associated with OS and PFS of BTC patients treated with 1^st^ line ICIs.

LIPI classification	Patients treated with the 1st line ICIs			
	OS (months)		PFS (months)	
	Median	HR (95% CI)	Median	HR (95% CI)
The good (*n* = 30)	34.43	1 [reference]	12.93	1 [reference]
The intermediate/poor (*n* = 49)	15.8	2.147 (1.061-4.344)	5.87	3.006 (1.601-5.642)
*P* value	0.03		<0.001	

PFS: progression-free survival; OS: overall survival; LIPI: lung immune prognostic index; BTC: biliary tract cancer; ICIs: immune checkpoint inhibitors.

**Table 7:** Univariate analyses of LIPI associated with OS and PFS of BTC patients treated with ICIs in subsequent lines.

LIPI classification	Patients treated with ICIs in subsequent lines			
	OS (months)		PFS (months)	
	Median	HR (95% CI)	Median	HR (95% CI)
The good (*n* = 8)	34.43	1 [reference]	12.93	1 [reference]
The intermediate/poor (*n* = 23)	15.8	1.140 (0.457-2.844)	5.87	1.505 (0.638-3.552)
*P* value	0.778		0.345	

PFS: progression-free survival; OS: overall survival; LIPI: lung immune prognostic index; BTC: biliary tract cancer; ICIs: immune checkpoint inhibitors.

## Data Availability

The raw data supporting the conclusions of this article will be made available by the authors, without undue reservation.

## Abbreviations

BTC: Biliary tract cancer
CCA: Cholangiocarcinoma
MDT: Multidisciplinary Treatment
GP: Gemcitabine and cisplatin
OS: Overall survival
ICIs: Immune checkpoint inhibitors
LIPI: Lung immune prognostic index
LDH: Lactate dehydrogenase
dNLR: Derived neutrophil-to-lymphocyte
NSCLC: Non-small-cell lung cancer
ECOG PS: Eastern Cooperative Oncology Group performance status scores
TRAEs: Treatment-related adverse events
WBC: White blood cell count
ANC: Absolute neutrophil count
ALC: Absolute lymphocyte count
DCR: Disease control rate
ORR: Overall response rate
CR: Complete response
PR: Partial response
SD: Stable disease
PFS: Progression-free survival
ROC: Receiver operator characteristic
PD: Progressive disease
HPD: Hyperprogressive disease
PD-L1: Programmed death ligand-1
TMB: Tumor mutational burden
MSI: Microsatellite steady-state
bTMB: Blood TMB
TAN: Tumor-associated neutrophils
NLR: Neutrophil-to-lymphocyte ratio
PLR: Platelet-lymphocyte ratio
Hb: Hemoglobin.

## Supplementary Materials

Supplementary Materials Supplementary 1: attached file 1A: sample calculation. Supplementary 2: Figure 1: PFS (a) and OS (b) according to LIPI groups of patients with advanced BTC treated with the ICIs.
