# Supplementary material for: Analysis of a Systemic Inflammatory Biomarker in Advanced Bile Tract Carcinoma Treated with Anti-PD-1 Therapy: Prognostic and Predictive Significance of Lung Immune Prognostic Index Score
Source: J Oncol. 2022 Mar 17;2022:1427779. doi: 10.1155/2022/1427779 (PMC8947875; doi:10.1155/2022/1427779)
Supplement: Supplementary Materials — Supplementary 1: attached file 1A: sample calculation. Supplementary 2: Figure 1: PFS (a) and OS (b) according to LIPI groups of patients with advanced BTC treated with the ICIs. [file 1427779.f1.zip › __ 1.pdf]

Figure1 ROC curve of pretreatment dNLR in assessment of the tumor progression rate at 6th month.

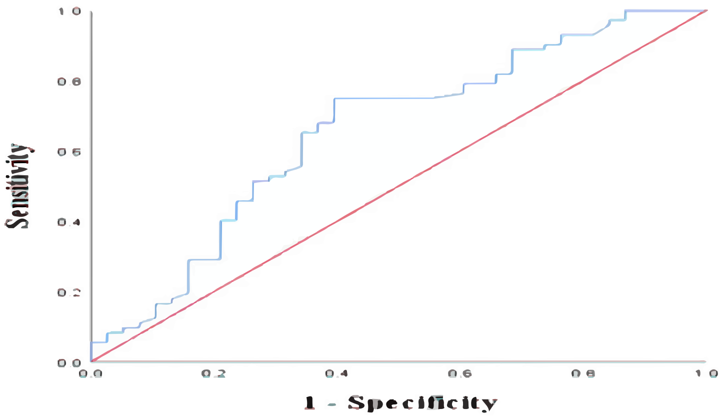

ROC: Receiver operator characteristic; dNLR: derived neutrophil-to-lym- phocyte
